# Influence of maternal vitamin D and calcium levels and psychoactive substance use during pregnancy on head circumference of newborns: cohort study, Rio de Janeiro, 2021-2022

**DOI:** 10.1590/S2237-96222026v35e20240911.en

**Published:** 2026-05-11

**Authors:** Danuza Gandra Rossi, Ana Lúcia Vollú, Ísis Midlej Carneiro Oliveira, Laura Guimarães Primo, Nataly Damasceno Figueiredo, Carmen Ildes Fróes Rodrigues Asmus, Gloria Fernanda Barbosa de Araújo Castro, Andréa Fonseca-Gonçalves

**Affiliations:** 1 Universidade Federal do Rio de Janeiro, Rio de Janeiro, RJ, Brazil Universidade Federal do Rio de Janeiro Rio de Janeiro RJ Brazil

**Keywords:** Pregnant People, Infant, Newborn, Vitamin D, Calcium, Cephalometry., Personas Embarazadas, Recién nacido, Vitamina D, Calcio, Cefalometría.

## Abstract

**Objective:**

To investigate the impact of maternal serum vitamin D and calcium levels and psychoactive substance use during pregnancy on the head circumference of newborns from a maternity hospital.

**Methods:**

Data were collected through interviews, laboratory tests, and medical records, involving 844 pregnant people. Exposure variables included vitamin D and calcium levels, alcohol consumption, tobacco, and drug use during pregnancy. The outcome was head circumference, assessed both as a continuous variable and as a categorical variable (<32 cm or ≥32 cm). Correlation tests (r), Kruskal-Wallis, Mann-Whitney, and regression analyses were used to estimate risks, with a significance level of 5%.

**Results:**

Of the pregnant people, 28.4% smoked, 41% consumed alcohol, and 4.6% used drugs; 90.3% had insufficient vitamin D, and 62.5% had below-normal calcium levels. Most deliveries were by cesarean section (57.3%), 16.1% were premature, 4% were twins, 51.1% were female, and 12.8% were low birth weight infants. There was a strong correlation between birth weight and head circumference (r 0.805; p-value<0.001), a weak correlation with calcium levels (r 0.129; p-value 0.01), and none with vitamin D levels (r 0.051; p-value 0.310). Infants of mothers who consumed alcohol (33.77±2.02 cm) and used drugs (33.32±1.72 cm) had smaller head circumferences (p-value<0.05) compared with those whose mothers did not (34.04±1.99 cm; 33.96±2.02 cm). Preterm birth, twin pregnancies, female sex, and low birth weight were associated with smaller head circumferences (p-value<0.05). No variable was associated with head circumference <32 cm (p-value>0.05).

**Conclusion:**

Infants of mothers who consumed alcohol, used drugs, and had low calcium levels had smaller head circumferences; however, no factor was associated with values below 32 cm.

## Resumen

**Objetivo:**

Investigar el impacto de los niveles séricos de vitamina D y calcio y del consumo de sustancias psicoactivas durante el embarazo sobre la circunferencia cefálica de recién nacidos en una maternidad.

**Métodos:**

Se recopilaron datos mediante entrevistas, análisis de laboratorio y revisión de historias clínicas, incluyendo 844 gestantes. Las variables de exposición incluyeron niveles de vitamina D, calcio y el consumo de alcohol, tabaco y drogas durante el embarazo. El desenlace fue la circunferencia cefálica, evaluada de manera continua y categórica (<32 cm o ≥32 cm). Se utilizaron pruebas de correlación (r), Kruskal-Wallis, Mann-Whitney y regresión para estimar riesgos, considerando un nivel de significancia del 5%.

**Resultados:**

Entre las gestantes, el 28,4% fumaba, el 41% consumía alcohol y el 4,6% usaba drogas; el 90,3% presentó deficiencia de vitamina D y el 62,5% niveles de calcio por debajo de lo normal. La mayoría de los partos fue por cesárea (57,3%), con un 16,1% de nacimientos prematuros, un 4% de gestaciones gemelares, 51,1% de niñas y 12,8% de recién nacidos con bajo peso. Se observó una fuerte correlación entre peso al nacer y circunferencia cefálica (r=0,805; valor de p<0,001), una correlación débil con los niveles de calcio (r=0,129; valor de p 0,01) y ninguna correlación con los niveles de vitamina D (r=0,051; valor de p 0,310). Los recién nacidos de madres que consumieron alcohol (33,77±2,02 cm) y drogas (33,32±1,72 cm) presentaron circunferencias menores (valor de p<0,05) en comparación con aquellos cuyas madres no consumieron (34,04±1,99 cm; 33,96±2,02 cm). La prematuridad, las gestaciones gemelares, el sexo femenino y el bajo peso se asociaron con circunferencias menores (valor de p<0,05). Ninguna variable se asoció con circunferencia cefálica <32 cm (valor de p >0,05).

**Conclusión:**

Los recién nacidos de gestantes que consumieron alcohol, drogas y presentaron bajos niveles de calcio mostraron circunferencias menores; sin embargo, ningún factor se asoció con valores inferiores a 32 cm.

**Ethical aspects**

This research respected ethical principles, having obtained the following approval data:

Research ethics committee: Universidade Federal do Rio de Janeiro

Opinion number: 4,676,124

Approval date: 28/4/2021

Certificate of submission for ethical appraisal: 44797721,3,0000,5275

Informed consent record: Obtained from all participants prior to data collection.

## Introduction 

Head circumference at birth is a significant indicator of fetal brain and overall development. Studies have shown that variations in this measure may result from alterations during intrauterine growth, potentially associated with maternal and environmental factors during pregnancy ^1^^-^^4^. There is growing evidence that head circumference at birth may serve as a proxy to assess fetal growth, influenced by multiple nutritional and environmental factors ^2^.

Among these, vitamin D and calcium deficiency during pregnancy have been recognized for their relevance in fetal bone metabolism and skeletal development ^5^^,^^6^. Vitamin D, in particular, regulates bone mineralization and may influence cranial growth; its deficiency in pregnant women has been associated with alterations in cranial bone formation ^7^. Moreover, adequate serum levels of calcium and phosphorus are crucial for fetal bone development, and deficiencies of these nutrients may compromise cranial growth, alter head circumference and potentially affect neurodevelopment ^8^.

Additionally, psychoactive substance use-including alcohol, illicit drugs, and tobacco-during pregnancy has been associated with restricted fetal growth, including reduced head circumference at birth ^9^. These factors may interfere with the supply of nutrients and oxygen to the fetus, increasing the risks of impaired growth and subsequent neurological alterations ^10^.

At birth, the average head circumference is about 35 cm, typically increasing by 1 cm per month during the first year of life. Term infants with a head circumference below 32 cm are diagnosed with reduced cranial size or microcephaly ^11^. There is evidence that infants with reduced head circumference and low birth weight exhibit a growth pattern associated with tissue alterations, including vascular changes ^12^. Furthermore, the mortality rate from ischemic heart disease among one-year-old individuals who had reduced head circumference and low birth weight was three times higher compared with those with normal weight and head circumference ^13^. Given the central role of head circumference as a marker of fetal growth, this birth cohort study aims to investigate whether maternal serum vitamin D and calcium levels, as well as alcohol consumption, drug and tobacco use during pregnancy, influence head circumference measurements in newborn infants. 

## Methods 

Study design and setting

This is a cohort study nested within the birth cohort of the Childhood and Environmental Pollutants Project (*Projeto Infância e Poluentes Ambientais*, PIPA) at the Federal University of Rio de Janeiro (UFRJ). Data from pregnant women and infants born at the University Maternity Hospital of the Federal University of Rio de Janeiro (*Maternidade Escola da Universidade Federal do Rio de Janeiro*, ME-UFRJ) were collected with signed informed consent from participants (pregnant people and legal guardians of the infants), from June 2021 to November 2022. Data collection included guided interviews using a structured questionnaire, review of infants' medical records, and laboratory analyses of pregnant individuals' blood samples, to investigate the associations between pregnant individuals' vitamin D and calcium levels, psychoactive substance use during pregnancy, and the mean head circumference of newborns at birth.

Participants and eligibility criteria

All pregnant people in the third trimester residing in the municipality of Rio de Janeiro, who had received prenatal care at the University Maternity Hospital of the Federal University of Rio de Janeiro (ME-UFRJ) during the study period and who consented to participate, were included. Their infants, born by vaginal or cesarean delivery (including forceps-assisted), at the same facility, with no gestational age restrictions and including twins, also participated in this study.

Pregnant people with occurrences of fetal death or spontaneous abortion were excluded. In addition, infants who tested positive for Zika virus serology or who were found to have craniofacial dysmorphism in the delivery room were also excluded.

Data sources and measurement

Gestational and newborn data were collected through interviews with pregnant people in the third trimester, from infants' medical records after birth, and from the pregnant people's laboratory tests. The outcome variable was the mean head circumference of newborn infants, expressed in centimeters and dichotomized (<32 cm or ≥32 cm).

The following exposure variables were investigated: maternal tobacco use (yes/no), illicit drug use (yes/no), and alcohol consumption (yes/no); and maternal serum calcium and vitamin D (25D) levels. Blood samples for measuring total calcium and vitamin D levels were collected in the third trimester of pregnancy. Vitamin D values were classified as insufficient (<30 ng/mL) or sufficient/normal (≥30 ng/mL). For total calcium, values were classified as below normal (<8.8 mg/dL) and normal (8.8-11.0 mg/dL).

Vitamin D and total calcium analyses were performed at the Clinical Analysis Laboratory of the Faculty of Pharmacy, Federal University of Rio de Janeiro. Serum 25-hydroxyvitamin D (ng/mL) was assessed using the electrochemiluminescence method ^14^, with the commercial kit Elecsys Vitamin D total III (Roche Diagnostics Corporation, Indianapolis, Indiana, USA); and total calcium (mg/dL) was measured by the Arsenazo III method ^15^, using a commercial kit (Labtest/Labmax Plenno Equipment, Buenos Aires, Argentina), with both tests using 750 µL of blood (plasma).

Infant sex (female/male), birth weight (<2500 g/≥2500 g), mother's age (years), type of pregnancy (singleton or twin), and gestational age (term/preterm) were considered predictor variables based on theoretical references, as they may be direct determinants of cranial growth ^12^^,^^13^. Maternal age was included due to its association with the risk of pregnancy complications. In addition, the following effect modifiers were also collected: infant race/skin color (White/Brown [Brazilian mixed race]/Black/Other), pregnant people's parity (primiparous: yes/no), and type of delivery (vaginal/Cesarean/forceps), as they may influence fetal growth ^16^^,^^17^. 

Sample size

For sample size calculation, the calculator from the University of São Paulo (http://estatistica.bauru.usp.br/calculoamostral/calculos.php) was used, and results from a previous study ^18^ were considered to assess the correlation between maternal serum calcium and vitamin D levels and the mean head circumference of newborn infants. Using Pearson's correlation data (r) (r=0.057 for calcium levels; p-value 0.332; and r=0.057 for vitamin D; p-value 0.333), assuming an alpha error (α) of 0.05 and a test power of 80%, 389 infants would be required to detect a possible correlation between vitamin D and calcium levels and mean head circumference values.

Differences in mean head circumference between infants of pregnant people exposed and unexposed to illicit drugs, alcohol, and tobacco were also estimated. Therefore, considering α=0.05, β=0.50, a minimum difference of 0.5 mm in mean head circumference between the exposed and unexposed groups for each exposure variable, and a maximum estimated standard deviation of 4.0, approximately 500 newborn infants would be required to observe differences in continuous mean head circumference values.

Furthermore, for multiple regression models, all pregnant person-infant pairs recruited for the PIPA-UFRJ cohort with at least one available value for the variables under investigation were included in the sample.

Statistical methods

Statistical analyses were performed using the Statistical Package for the Social Sciences (SPSS, version 24.0, Chicago, Illinois, USA) and included both descriptive and inferential investigations. Continuous data were analyzed using Pearson's correlation test (r) to evaluate correlations between mean head circumference and birth weight, maternal serum vitamin D levels, and serum calcium levels of newborn infants. Additionally, Kruskal-Wallis and Mann-Whitney tests were used to assess differences in continuous head circumference measurements, considering exposure variables (alcohol consumption, tobacco, and illicit drug use), predictor variables (infant sex, birth weight, maternal age, singleton or multiple pregnancy, and gestational age), and potential effect modifiers (infant race/ethnicity, maternal parity status [primiparous/multiparous], and type of delivery).

Associations between all categorical variables and head circumference (<32 cm/≥32 cm) were investigated using the chi-square test. Poisson regression models were used to estimate adjusted and unadjusted relative risks (RR) with 95% confidence intervals (CI). The statistical significance of the RR was determined using a robust variance estimator with a covariance matrix, evaluated by the Wald chi-square test. Variables with significance below 0.20 (p-value 0.20) were used as parameters to remain as adjustment variables in the multivariate models that tested the influence of exposure factors on head circumference measurements (<32 cm/≥32 cm; dependent variable). It is noteworthy that the analysis of continuous data allows for the detection of subtle associations with exposure factors, even in the absence of microcephaly, whereas analysis using categorical variables follows the Ministry of Health criteria for screening microcephaly in term newborn infants ^11^. This combined approach enables a more comprehensive assessment of neonatal cranial growth.

The level of statistical significance was set at 5%. 

## Results 

Of the 1,513 pregnant women eligible who attended the maternity hospital for prenatal care during the recruitment period, 901 were enrolled. Of these, 11 lived outside the municipality of Rio de Janeiro and 46 had their babies in other hospitals. No fetal deaths or spontaneous abortions were observed. Thus, 844 pregnant people and their newborn infants participated in the study (Figure 1). Among the infants tested for Zika virus serology (n=535), none showed positive results. The other 309 infants who were not tested for the Zika virus did not present craniofacial dysmorphism. Therefore, no infant was excluded for these reasons. The mean age of the pregnant people at the time of delivery was 29.3±6.8 years. Among the pregnant people, 293 (36.2%) were primiparous, 34 (4.0%) had twin pregnancies, 136 (16.1%) had preterm deliveries, and most underwent cesarean section (n=482, 57.2%). During pregnancy, 240 (28.4%) smoked (tobacco), 332 (41.0%) consumed alcohol, and 37 (4.6%) used illicit drugs. Regarding newborn infants' characteristics, 431 (51.1%) were female, 280 (37.6%) were classified as brown, and 107 (12.8%) had low birth weight (Table 1). In addition, the mean head circumference was 33.85±2.04 cm, and the incidence of head circumference <32 cm was 8.9% (73/821).

**Figure 1:**
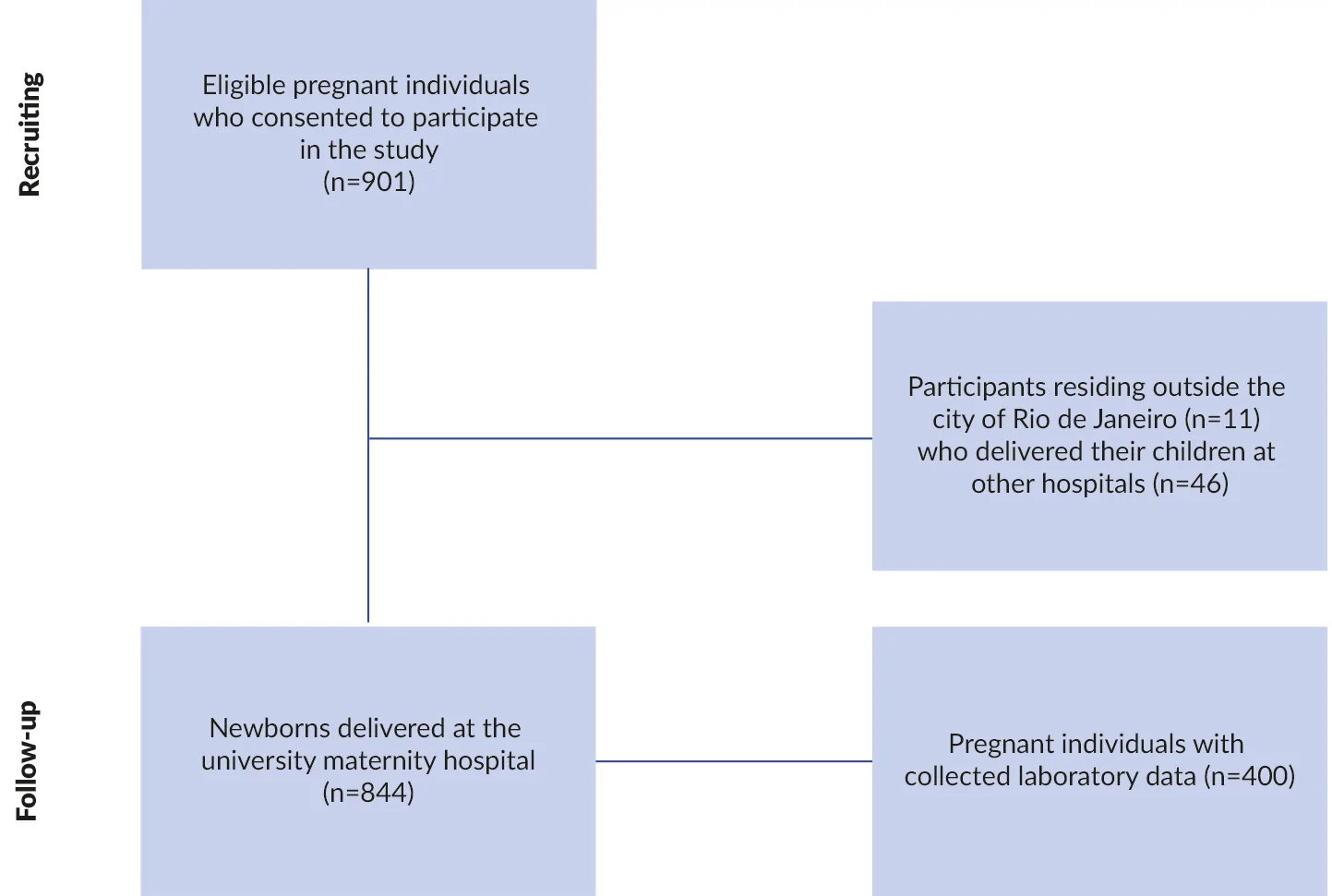
Demonstration of the recruitment and follow-up process of participating pregnant people and their newborn infants. University Maternity Hospital of the Federal University of Rio de Janeiro, Rio de Janeiro, 2021-2022 (n=844)

Considering serum calcium and vitamin D levels in the 400 pregnant people who had sufficient blood samples for these analyses, the following means were observed: calcium, 8.58±0.62 mg/dL; vitamin D, 20.10±7.52 ng/mL. Of this group, 361 (90.3%) had insufficient vitamin D levels, while 250 (62.5%) had calcium levels below normal (Table 1).

Although smaller head circumference values were associated with lower birth weight and lower serum calcium levels, the correlations were strong (r=0.805; p-value<0.001) and weak (r=0.129; p-value 0.01), respectively. For vitamin D levels, no correlation was found (r=0.051; p-value 0.310).

Lower mean head circumference values were observed among newborn infants whose mothers consumed alcohol (p-value 0.016) and illicit drugs (p-value 0.013) during pregnancy. Female, preterm, twin infants, and those with low birth weight also showed lower mean head circumference values. Table 2 presents the differences in mean head circumference according to each variable studied.

When the dichotomous outcome investigated (head circumference <32 cm/≥32 cm) was considered, female infants, preterm births, twins, and infants with low birth weight were associated with head circumference below 32 cm (Table 3). Therefore, these variables were included in the final multiple regression models (Table 4).

It was observed that none of the exposure factors were associated with head circumference below 32 cm (microcephaly) in any of the adjusted models (Table 4).

**Table 1:** Absolute (n) and relative (%) frequencies of the characteristics of the study's infants and pregnant people. University Maternity Hospital of the Federal University of Rio de Janeiro, Rio de Janeiro, 2021-2022

Variables	n (%)
Sex of infants (n=844)	
Female	431 (51.1)
Male	411 (48.7)
Undeterminedª	2 (0.2)
Infant race/skin color (n=745)	
White	243 (32.6)
Brown	280 (37.6)
Black	216 (29.0)
Other	6 (0.8)
Birth weight (n=833)	
<2,500 g	107 (12.8)
≥2,500 g	726 (87.2)
Head circumference (n=821)	
<32 cm	73 (8.9)
≥32 cm	748 (91.1)
Alcohol consumption (n=809)	
Yes	332 (41.0)
No	477 (59.0)
Tobacco use (n=844)	
Yes	240 (28.4)
No	604 (71.6)
Drug use (n=807)	
Yes	37 (4.6)
No	770 (95.4)
Type of birth (n=843)	
Vaginal	359 (42.5)
Cesarean section	482 (57.3)
Forceps	2 (0.2)
Type of pregnancy (n=844)	
Singleton	810 (96.0)
Twin	34 (4.0)
Gestational age (n=844)	
Term birth	708 (83.9)
Preterm	136 (16.1)
Primiparous (n=809)	
Yes	293 (36.2)
No	516 (63.8)
Calcium levels (n=400)	
Below	250 (62.5)
Vaginal	150 (37.5)
Vitamin D levels (n=400)	
Sufficient	39 (9.7)
Insufficient	361 (90.3)

ªSex was not identified in the delivery room and was classified as undetermined.

**Table 2:** Difference between means (values in millimeters) and respective standard deviations (SD) of newborn infants' head circumference, according to participants' variables. University Maternity Hospital of the Federal University of Rio de Janeiro, Rio de Janeiro, 2021-2022

Variables	Head circumference (mean±SD)	p-value
Sex of infantª		<0.001
Female	33.53±2.15	
Male	34.22±1.82	
Race/skin color		0.747
White	33.97±1.89	
Brown	33.84±2.16	
Black	34.01±1.89	
Other	34.58±1.56	
Birth weight		<0.001
<2,500 g	30.47±2.40	
≥2,500 g	34.35±1.41	
Alcohol consumption		0.016
Yes	33.77±2.02	
No	34.04±1.99	
Use of tobacco		0.192
Yes	33.74±2.03	
No	33.89±2.04	
Illicit drug use		0.013
Yes	33.32±1.72	
No	33.96±2.02	
Type of delivery		0.196
Vaginal	33.71±1.80	
Cesarean section	33.96±2.19	
Forceps	33.50±0.70	
Type of pregnancy		<0.001
Singleton	33.95±1.98	
Twin	31.45±2.09	
Gestational age		<0.001
Term birth	34.30±1.50	
Preterm	31.39±2.68	
Primiparous		0.151
Yes	33.79±1.88	
No	34.00±2.08	

ªThe head circumference values of infants with indeterminate sex were not included in the statistical analyses.

**Table 3:** Association between infant head circumference (<32 cm or ≥32 cm) with study variables, bivariate analysis, relative risk (RR), and 95% confidence intervals (95%CI). University Maternity Hospital of the Federal University of Rio de Janeiro, Rio de Janeiro, 2021-2022

Variable		Head circumference			Poisson regression		
	n	<32 cm	≥32 cm	p-value	Unadjusted model		
		n (%)	n (%)		RR	95%CI	p-value
Sex of infant				0.006			
Male	406	25 (3.0)	381 (46.5)		1.056	(1.016; 1.098)	0.006
Female	414	48 (5.9)	366 (44.6)		1		
Race/skin color				0.816			
Other	6	-	6 (0.8)		-	-	-
Brown	275	23 (3.2)	252 (34.6)		0.980	(0.937; 1.026)	0.391
Black	212	16 (2.2)	196 (26.9)		0.988	(0.943; 1.036)	0.630
White	235	15 (2.1)	220 (30.2)		1		
Birth weight				<0.001			
≥2,500 g	721	12 (1.5)	709 (86.4)		1.810	(1.644; 1.993)	<0.001
<2,500 g	100	61 (7.4)	39 (4.8)		1		
Alcohol consumption				0.414			
No	467	33 (4.2)	434 (54.9)		1.016	(0.978; 1.056)	0.422
Yes	324	28 (3.5)	296 (37.4)		1		
Use of tobacco				0.359			
No	589	49 (6.0)	540 (65.8)		1.020	(0.975; 1.068)	0.379
Yes	232	24 (2.9)	208 (25.3)		1		
Use of drugs				0.189			
No	753	56 (7.1)	697 (88.3)		1.067	(0.951; 1.196)	0.269
Yes	36	5 (0.6)	31 (3.9)		1		
Type of delivery				1.000			
Vaginal	353	31 (3.8)	322 (39.3)		1.003	(0.964; 1.043)	0.901
Cesarean section	465	42 (5.1)	423 (51.6)		1		
Forceps	2	-	2 (0.2)		-	-	-
Type of pregnancy				<0.001			
Twin	30	14 (1.7)	16 (1.9)		0.676	(0.565; 0.808)	<0.001
Singleton	791	59 (7.2)	732 (89.2)		1		
Gestational age				<0.001			
Term birth	696	21 (2.6)	675 (82.2)		1.471	(1.348; 1.605)	<0.001
Preterm	125	52 (6.3)	73 (8.9)		1		
Primiparous				0.971			
No	504	39 (4.9)	465 (58.8)		0.999	(0.961; 1.039)	0.971
Yes	287	22 (2.8)	265 (33.5)		1		
Calcium levels				0.577			
Below	244	26 (6.7)	218 (55.9)		0.983	(0.925; 1.044)	0.569
Vaginal	146	13 (3.3)	133 (34.1)		1		
Vitamin D levels				0.955			
Insufficient	351	35 (9.0)	316 (81.0)		1.003	(0.907; 1.109)	0.956
Sufficient	39	4 (1.0)	35 (9.0)		1		

**Table 4:** Influence of exposure factors on mean infant head circumference (<32 cm/≥32 cm), as shown by multiple models adjusted^a^ for: birth weight (Model 0); sex (Model 1); type of delivery (Model 2); and gestational age (Model 3). University Maternity Hospital of the Federal University of Rio de Janeiro, Rio de Janeiro, 2021-2022

	Model 0					Model 1				
	Total	n	RR	(95%CI)	p-value	Total	n	RR	(95%CI)	p-value
Vitamin D<30 ng/mL	390	351	0.981	(0.922; 1.044)	0.543	390	351	1.004	(0.910; 1.109)	0.931
Calcium<8.8 mg/dL	390	244	0.975	(0.934; 1.019)	0.263	390	244	0.990	(0.933; 1.052)	0.753
No alcohol consumption	791	467	1.006	(0.977; 1.035)	0.699	790	467	1.015	(0.977; 1.055)	0.444
No tobacco use	821	589	1.025	(0.990; 1.061)	0.158	820	589	1.019	(0.974; 1.066)	0.412
No drug use	789	753	1.060	(0.969; 1.160)	0.202	788	752	1.065	(0.950; 1.194)	0.280
	Model 2					Model 3				
	Total	n	RR	(95%CI)	p-value	Total	n	RR	(95%CI)	p-value
Vitamin D<30 ng/mL	390	351	1.022	(0.926; 1.129)	0.662	390	351	1.007	(0.937; 1.083)	0.844
Calcium<8.8 mg/dL	390	244	0.971	(0.918; 1.027)	0.304	390	244	0.982	(0.931; 1.037)	0.523
No alcohol consumption	791	467	1.010	(0.973; 1.049)	0.601	791	467	1.017	(0.983; 1.053)	0.351
No tobacco use	821	589	1.042	(0.997; 1.089)	0.069	821	589	1.038	(0.999; 1.079)	0.059
No drug use	789	753	1.069	(0.954; 1.199)	0.251	789	753	1.098	(0.983; 1.226)	0.097

a Reference categories**:** Vitamin D ≥30 ng/mL, Calcium=8.8-11.0 mg/dL, alcohol use, tobacco use, illicit drug use; Model 0: adjusted for birth weight; Model 1: adjusted for sex, reference category: female; Model 2: adjusted for type of delivery, reference category: singleton delivery; Model 3: adjusted for gestational age, reference category: term delivery. Reference for the outcome: head circumference ≥32 cm. Relative risk (RR); 95% confidence interval (95%CI).

## Discussion 

Among the main findings of the present study, it was observed that infants born to mothers who consumed alcohol and illicit drugs, female infants, preterm infants, twins, and those with low birth weight had lower head circumference values. In addition, a strong positive correlation was found between infant weight and head circumference. Previous studies ^9^^,^^20^^,^^21^ have also indicated that alcohol consumption and drug use during pregnancy may negatively affect fetal development, including head circumference, which supports the findings of this study. Furthermore, preterm birth and low birth weight are widely recognized as factors that negatively influence cranial growth ^21^. However, although lower mean head circumference values were observed in infants born to mothers who consumed alcohol and used illicit drugs during pregnancy, no association was observed between microcephaly (head circumference <32 cm) and such exposure factors, nor with the other factors investigated. Since the mean head circumference value in the studied sample was above 32 cm and the incidence of microcephaly was 8.9% (73/821), it is suggested that these results may have influenced the lack of association between exposure factors and the outcome investigated. Additionally, the possibility that environmental or nutritional factors not considered in the analysis may have contributed to the absence of association. 

Regarding tobacco use during pregnancy, the results presented in this study indicate that there was no statistically significant association with newborn infants' head circumference values. Nevertheless, there is evidence that smoking during pregnancy is inversely associated with infant weight, length, and head circumference at birth ^19^. One limitation of the present study is precisely the fact that mothers were not asked about the frequency and number of cigarettes smoked, the duration of tobacco exposure, or the trimester of pregnancy in which the highest consumption occurred-crucial factors for assessing the effects of smoking on fetal development, which may have influenced the results obtained. Even so, it is important to highlight that we observed a slight trend (p-value 0.059) toward an association between nonsmoking pregnant people and normal head circumference values (≥32 cm) in cases of preterm delivery (Table 4). It is known that newborn infants of women who continued smoking after the first trimester had a smaller head circumference compared to those whose mothers quit smoking ^20^. Thus, we believe that further studies are needed to clarify whether mean head circumference values, especially among preterm infants, become even lower when pregnant people smoke during pregnancy. It is also important to note that the use of such psychoactive substances may compromise the supply of nutrients and oxygen to the fetus, affecting its growth and neurological development through pathways involving placental and systemic alterations.

Consistent with a previous study ^22^, although differing in the method of micronutrient assessment, the present study observed an alarming situation regarding vitamin D deficiency and low calcium levels among the participating mothers. This deficiency in a large part of the sample highlights the magnitude of the problem among pregnant people, raising concerns about maternal and fetal health. It is worth noting that the mothers were monitored throughout their pregnancy through prenatal medical care. In addition, laboratory tests to assess vitamin D and calcium levels are not commonly requested for pregnant women receiving care through the Brazilian National Health System (*Sistema Único de Saúde*, SUS), which raises concerns about the importance of including these tests in prenatal care. We highlight that the sample may have been subject to selection bias, as participants with access to specific health services or a greater interest in prenatal care may have been disproportionately represented. Moreover, not all pregnant people assessed had their serum vitamin D and calcium levels measured. This restriction may have introduced selection bias, considering that those who underwent testing may differ from those who did not, potentially influencing the results. We also recognize that micronutrient exposure was assessed using a single point measurement, which may not reflect variations across pregnancy. These limitations may influence the generalization of the results.

It should also be considered that the absence of ionized calcium measurement-the physiologically active form-may have implications for the interpretation of our findings. Since only total calcium was measured, which includes both free and protein-bound calcium, it was not possible to distinguish variations reflecting bioavailable calcium. This limitation affects the accuracy of the calcium assessment in participants and may reduce sensitivity in detecting true associations between calcium levels and the factors studied. Therefore, the results of this study should be interpreted with caution, recognizing that total calcium measurement provides an overall view but may not fully reflect the physiological status of calcium in the pregnant people evaluated. For future research, a more robust assessment of calcium status would be advisable.

The authors emphasize that individuals with darker skin have natural protection against sunlight, as melanin absorbs UVB radiation, and require three to five times more sun exposure to synthesize the same amount of vitamin D as those with lighter skin ^23^. In the present study, most infants were classified as Brown or Black, with their mothers exhibiting similar characteristics. This may have contributed to the results observed, given that vitamin D synthesis depends on sun exposure. Another possible reason could be inadequate dietary intake, as suggested in a study ^22^ that found low consumption of essential nutrients, such as calcium and vitamin D, among pregnant women from two cohorts, regardless of gestational trimester. Furthermore, the investigation emphasized that, regardless of race/skin color and vitamin supplementation, promoting a balanced diet should be a priority from the beginning of prenatal care and maintained throughout pregnancy. In this sense, further studies are needed to jointly evaluate diet, sun exposure, and serum vitamin D levels in pregnant people.

The positive correlation observed in this study between lower maternal serum calcium levels and smaller head circumference measurements, although weak, suggests that maternal mineral metabolism may affect infant bone development, corroborating previous research ^8^. However, regarding serum vitamin D levels, no correlation was found with the mean head circumference of newborn infants, in contrast to some studies that indicate vitamin D insufficiency as a critical factor in fetal bone development ^24^^-^^26^. The insufficiency of essential nutrients, such as calcium and phosphorus, combined with demographic factors (age, sex, and race/skin color) and environmental factors (sun exposure), may modulate vitamin D deficiency and its biological effects ^27^. Such complex interactions can influence bone mineralization and, consequently, mask the effects of vitamin D on cranial growth. The absence of a correlation found in this study does not invalidate the role of vitamin D in fetal development, but underscores the need for further investigations to elucidate the complexity of these relationships. 

The findings of the present study are relevant and highlight an important gap in prenatal care, as identifying risk factors is crucial for formulating effective preventive strategies and providing support during pregnancy. Therefore, although the head circumference values observed were not classified as infants with microcephaly, this study highlights that these measurements were lower in newborn infants whose mothers consumed alcohol and used illicit drugs during pregnancy. Previous studies ^28^^,^^29^ suggest that a smaller head circumference at birth may be associated with an increased risk of later cranial asymmetries, emphasizing the relevance of this measure as an early marker of cranial growth and morphology. Furthermore, the correlation observed between maternal calcium levels and smaller infant head circumference, although weak, underscores the importance of adequate prenatal care and preventive measures during pregnancy.

## Data Availability

The database and the analysis codes used in the research are available by accessing the link: https://data.scielo.org/dataverse/scielodata?q=&fq1=publicationStatus%3A%22Draft%22&fq0=dvObjectType%3A%28dataverses+OR+datasets%29&types=dataverses%3Adatasets&sort=dateSort&order=.
