# Supplementary material for: Influence of maternal vitamin D and calcium levels and psychoactive substance use during pregnancy on head circumference of newborns: cohort study, Rio de Janeiro, 2021-2022
Source: Epidemiol Serv Saude. 2026 May 11;35:e20240911. doi: 10.1590/S2237-96222026v35e20240911.en (PMC13686384; doi:10.1590/S2237-96222026v35e20240911.en)
Supplement: Translation

# Translation: Influência dos níveis maternos de vitamina D e de cálcio e do uso de substâncias psicoativas na gestação sobre o perímetro cefálico de recém-nascidos: estudo de coorte, Rio de Janeiro, 2021-2022

**DOI:** 10.1590/S2237-96222026v35e20240911.pt

**Authors:** Danuza Gandra Rossi, Ana Lúcia Vollú, Ísis Midlej Carneiro Oliveira, Laura Guimarães Primo, Nataly Damasceno Figueiredo, Carmen Ildes Fróes Rodrigues Asmus, Gloria Fernanda Barbosa de Araújo Castro, Andréa Fonseca-Gonçalves

**Affiliations:** 1 Universidade Federal do Rio de Janeiro, Rio de Janeiro, RJ, Brasil Universidade Federal do Rio de Janeiro Rio de Janeiro RJ Brasil

**Keywords:** Gestantes, Recém-nascido, Vitamina D, Cálcio, Perímetro Cefálico

## Abstract

**Objetivo:**

Investigar o impacto dos níveis séricos de vitamina D e de cálcio e do uso de substâncias psicoativas durante a gestação no perímetro cefálico de recém-nascidos de uma maternidade.

**Métodos:**

Coletaram-se dados por entrevistas, exames laboratoriais e prontuários, envolvendo 844 gestantes. As variáveis de exposição incluíram níveis de vitamina D, de cálcio e uso de álcool, tabaco e drogas na gestação. O desfecho foi o perímetro cefálico, avaliado como contínuo e categórico (<32 cm ou ≥32 cm). Utilizaram-se testes de correlação (r), Kruskal-Wallis, Mann-Whitney e de regressão para estimar riscos, com significância de 5%.

**Resultados:**

Das gestantes, 28,4% fumavam, 41% consumiam álcool e 4,6% usavam drogas, 90,3% apresentavam vitamina D insuficiente e 62,5% cálcio abaixo do normal. A maioria dos partos foi através de cesariana (57,3%), sendo 16,1% prematuros, 4% de gêmeos, 51,1% de meninas e 12,8% de bebês com baixo peso. Houve forte correlação entre peso ao nascer e perímetro cefálico (r=0,805; p-valor<0,001), correlação fraca com os níveis de cálcio (r=0,129; p-valor 0,01) e nenhuma com os níveis de vitamina D (r=0,051; p-valor 0,310). Bebês de mães que consumiram álcool (33,77±2,02 cm) e drogas (33,32±1,72 cm) apresentaram perímetros menores (p-valor<0,05) comparados aos de mães que não usaram (34,04±1,99 cm; 33,96±2,02 cm). Prematuridade, gestações gemelares, sexo feminino e baixo peso associaram-se a perímetros menores (p-valor<0,05). Nenhuma variável associou-se ao perímetro cefálico<32 cm (p-valor>0,05).

**Conclusão:**

Bebês de gestantes que usaram álcool, drogas e tiveram baixos níveis de cálcio apresentaram perímetros menores, porém, nenhum fator associou-se a valores abaixo de 32 cm.

**Aspectos éticos**

Esta pesquisa respeitou os princípios éticos, obtendo os seguintes dados de aprovação:

Comitê de ética em pesquisa: Universidade Federal do Rio de Janeiro

Número do parecer: 4.676.124

Data de aprovação: 28/4/2021

Certificado de apresentação de apreciação ética: 44797721.3.0000.5275

Registro do consentimento livre e esclarecido: Obtido de todos os participantes antes da coleta.

## Introdução 

O perímetro cefálico ao nascimento destaca-se como um importante indicador de desenvolvimento cerebral e geral do feto. Estudos demonstram que variações nessa medida podem ser decorrentes de alterações, durante o crescimento intrauterino, potencialmente associadas a fatores maternos e ambientais durante a gestação ^1^^-^^4^. Há evidências crescentes de que o perímetro cefálico ao nascimento pode servir como proxy para avaliar o crescimento fetal, influenciado por diversos fatores nutricionais e ambientais ^2^.

Dentre esses fatores, a deficiência de vitamina D e de cálcio durante a gestação tem sido reconhecidas por sua relevância no metabolismo ósseo fetal e no desenvolvimento do esqueleto ^5^^,^^6^. A vitamina D, em particular, regula a mineralização óssea e pode afetar o crescimento craniano, tendo sido sua deficiência em gestantes relacionada a alterações na formação de ossos cranianos ^7^. Além disso, níveis séricos adequados de cálcio e de fósforo são essenciais para o desenvolvimento ósseo fetal, e déficits dessas substâncias podem comprometer o crescimento craniano, alterando o perímetro cefálico, o que é refletido em possível impacto neurodesenvolvimental ^8^.

Adicionalmente, o uso de substâncias psicoativas, como álcool, drogas ilícitas e tabaco, durante a gestação, tem sido associado à restrição do crescimento fetal, incluindo-se redução do perímetro cefálico ao nascimento ^9^. Esses fatores podem interferir na oferta de nutrientes e de oxigênio ao feto, agravando riscos de crescimento insuficiente e de alterações neurológicas posteriores ^10^.

Ao nascer, um bebê possui perímetro cefálico médio de 35 cm, que normalmente aumenta em um cm por mês durante o primeiro ano. Crianças não prematuras com perímetro cefálico inferior a 32 cm são diagnosticadas com tamanho craniano reduzido ou microcefalia ^11^. Existe evidência de que bebês com perímetro cefálico reduzido e baixo peso apresentam um padrão de crescimento associado a alterações teciduais, incluindo os vasos sanguíneos ^12^. Além disso, a taxa de mortalidade por doença cardíaca isquêmica entre indivíduos com um ano de idade que apresentaram perímetro cefálico reduzido e baixo peso foi três vezes maior em comparação àqueles com peso e perímetro cefálico normais ^13^. Diante do papel central do perímetro cefálico como um marcador do crescimento fetal, este estudo, por meio de uma coorte de nascimento, visa investigar se os níveis séricos de vitamina D e de cálcio nas gestantes, bem como o uso de álcool, drogas e tabaco durante a gestação influenciam nas medidas do perímetro cefálico de recém-nascidos. 

## Métodos 

Delineamento do estudo e contexto

Este é um estudo de coorte associado à coorte de nascimento do Projeto Infância e Poluentes Ambientais (PIPA) da Universidade Federal do Rio de Janeiro. Assim, dados de gestantes e de bebês nascidos na Maternidade Escola da Universidade Federal do Rio de Janeiro foram coletados com o consentimento assinado dos participantes (gestantes e responsáveis legais pelos bebês), de junho de 2021 a novembro de 2022, por meio de entrevistas guiadas com um questionário, anotações dos prontuários médicos dos bebês e análises laboratoriais de amostras de sangue das gestantes, a fim de investigar associações dos níveis de vitamina D e de cálcio da gestante e do uso de substâncias psicoativas durante a gestação com o valor médio do perímetro cefálico dos bebês ao nascer.

Participantes e critérios de elegibilidade

Foram incluídas todas as gestantes no terceiro período de gestação que moravam no município do Rio de Janeiro, que haviam recebido atendimento pré-natal na Maternidade Escola da Universidade Federal do Rio de Janeiro durante o período do estudo e que consentiram participar da pesquisa. Também participaram desta pesquisa os seus bebês, nascidos por parto vaginal ou cesáreo (mesmo com o auxílio de fórceps) no mesmo local, sem limites de idade gestacional, incluindo gêmeos.

Gestantes com ocorrências de morte fetal e de aborto espontâneo foram excluídas. Além disso, também foram excluídos bebês que foram testados e apresentaram sorologia positiva para o vírus Zika ou que foram examinados na sala de parto com dismorfia craniofacial.

Fontes de dados e variáveis

Os dados gestacionais e de nascimento do recém-nascido foram coletados por meio de entrevistas com as gestantes no terceiro trimestre da gestação, dos prontuários médicos dos bebês após o nascimento e por exames laboratoriais da gestante. A variável de desfecho foi a média do perímetro cefálico dos recém-nascidos, representado numericamente em centímetros e de forma dicotomizada (valores<32 cm ou ≥32 cm).

As seguintes variáveis de exposição foram investigadas: o uso materno de tabaco (sim/não), drogas ilícitas (sim/não) e álcool (sim/não); e os níveis séricos de cálcio e de vitamina D (25D) das gestantes. As amostras de sangue para detectar os níveis de cálcio total e de vitamina D foram coletadas no terceiro trimestre da gestação. Os valores de vitamina D foram considerados insuficientes (<30 ng/mL) ou suficientes/normais (≥30 ng/mL). Para o cálcio total, os valores foram estimados como abaixo do normal (<8,8 mg/dL) e normais (8,8-11,0 mg/dL).

As análises de vitamina D e de cálcio total foram realizadas no Laboratório de Análises Clínicas da Faculdade de Farmácia da Universidade Federal do Rio de Janeiro. A vitamina D 25-hidroxila (ng/mL) foi avaliada por meio do método de quimioluminescência eletroquímica ^14^, utilizando o kit comercial Elecsys Vitamin D total III (Roche Diagnostics Corporation, Indianápolis, Indiana, Estados Unidos); e o cálcio total (mg/dL) pelo método de arsenazo III ^15^, empregando kit comercial (Labtest/Labmax Plenno Equipment, Buenos Aires, Argentina), ambos a partir de alíquotas de 750 µL de sangue (plasma).

Sexo do bebê (feminino/masculino), peso ao nascer (<2500 g/≥2500 g), idade da mãe (em anos), tipo de gestação (única ou gemelar) e idade gestacional (a termo/prematuro) foram variáveis consideradas como preditoras com base em referências teóricas, uma vez que podem ser determinantes diretas do crescimento craniano ^12^^,^^13^. A idade materna foi considerada devido à sua associação com o risco de complicações gestacionais. Além disso, as seguintes variáveis modificadoras de efeito também foram coletadas: raça/cor da pele do bebê (branca/parda/negra/outras), tipo de gestante (status de primípara) (sim/não) e tipo de parto (normal/cesariana/fórceps), pois podem influenciar o crescimento fetal ^16^^,^^17^. 

Tamanho da amostra

Para o cálculo do tamanho da amostra, foi utilizada calculadora da Universidade de São Paulo (http://estatistica.bauru.usp.br/calculoamostral/calculos.php), e os resultados de estudo prévio ^18^ foram considerados para verificar a correlação entre os níveis séricos de cálcio e de vitamina D nas gestantes com os valores médios de perímetro cefálico dos bebês. Assim, utilizando-se os dados da correlação de Pearson (r) (r=0,057 para os níveis de cálcio; p-valor 0,332; e r=0,057 para vitamina D; p-valor 0,333), assumindo um erro alfa (α) de 0,05 e um poder do teste de 80%, seriam necessários 389 bebês para verificar uma possível correlação entre os níveis de vitamina D e de cálcio com os valores médios de perímetro cefálico.

Também foram estimadas as diferenças entre a média do perímetro cefálico dos bebês de gestantes expostas e não expostas a drogas ilícitas, álcool e tabaco. Assim, considerando um α=0,05, β=0,50 e uma diferença mínima de 0,5 mm entre a média do perímetro cefálico dos grupos expostos e dos não expostos a cada variável de exposição, além de uma estimativa máxima de desvio padrão de 4,0, seriam necessários 500 recém-nascidos para observar a diferença entre os valores médios contínuos de perímetro cefálico.

Além disso, para os modelos de regressão múltipla, foram incluídos na amostra todos os pares gestante-bebê recrutados para a coorte PIPA-UFRJ que possuíam pelo menos uma informação das variáveis investigadas.

Métodos estatísticos

As análises estatísticas foram realizadas utilizando o software Statistical Package for the Social Sciences (SPSS, versão 24.0, Chicago, Illinois, Estados Unidos), sendo submetidas à investigação descritiva e inferencial. Os dados contínuos foram analisados por meio do teste de correlação de Pearson (r), que foi utilizado para avaliar as correlações entre os valores médios do perímetro cefálico e o peso, os níveis séricos de vitamina D e os níveis séricos de cálcio dos recém-nascidos. Além disso, os testes de Kruskal-Wallis e de Mann-Whitney foram utilizados para observar diferenças nas medições contínuas de perímetro cefálico, considerando-se as variáveis de exposição (uso de álcool, tabaco e drogas ilícitas), as variáveis preditoras (sexo, peso ao nascer, idade da mãe, tipo de gestação e idade gestacional) e as variáveis modificadoras de efeito (raça/cor da pele do bebê, status de primípara ou multípara das gestantes e tipo de parto).

Foi investigada a associação entre todas as variáveis categóricas e o perímetro cefálico (<32 cm/≥32 cm) pelo teste qui-quadrado. Modelos de regressão de Poisson foram utilizados para estimar os riscos relativos (RR) brutos e ajustados e os seus intervalos de confiança de 95%. A significância estatística do RR foi obtida utilizando-se um estimador robusto de variância com matriz de covariância, avaliada pelo teste qui-quadrado de Wald. Variáveis com significância inferior a 0,20 (p-valor 0,20) foram os parâmetros utilizados para permanecer como variáveis de ajuste nos modelos multivariados que testaram a influência dos fatores de exposição nas medidas de perímetro cefálico (<32 cm/≥32 cm; variável dependente). Acrescenta-se que a análise dos dados contínuos permite identificar associações sutis com os fatores de exposição, mesmo na ausência de microcefalia, enquanto a análise com a variável categórica seguiu os critérios do Ministério da Saúde para rastreio de microcefalia em recém-nascidos a termo ^11^. Essa abordagem combinada possibilita uma avaliação mais abrangente do crescimento craniano neonatal.

O nível de significância estatística adotado foi de 5%. 

## Resultados 

Das 1.513 gestantes elegíveis que compareceram à maternidade para acompanhamento pré-natal durante o período de recrutamento, 901 foram inscritas. Destas, 11 moravam fora do município do Rio de Janeiro e 46 tiveram seus bebês em outros hospitais. Não foram observados óbitos fetais, nem abortos espontâneos. Assim, 844 gestantes e seus recém-nascidos participaram do estudo (Figura 1). Dos bebês que foram testados para a sorologia do vírus Zika (n=535), nenhum apresentou resultados positivos. Os outros 309 bebês que não foram testados para o vírus Zika não apresentaram dismorfia craniofacial. Assim, nenhum bebê foi excluído por esses motivos. A idade média das gestantes no momento do parto foi de 29,3±6,8 anos. Entre as gestantes, 293 (36,2%) eram primíparas, 34 (4,0%) estavam grávidas de gêmeos, 136 (16,1%) tiveram partos prematuros e a maioria passou por cesariana (n=482, 57,2%). Durante a gestação, 240 (28,4%) fumaram (tabaco), 332 (41,0%) consumiram álcool e 37 (4,6%) usaram drogas ilícitas. Quanto às características dos recém-nascidos, 431 (51,1%) eram meninas, 280 (37,6%) eram pardos e 107 (12,8%) apresentaram baixo peso ao nascer (Tabela 1). Além disso, o valor médio do perímetro cefálico foi de 33,85±2,04 cm, e a incidência de perímetro cefálico<32 cm foi de 8,9% (73/821).

**Figura 1:** Do processo de recrutamento e de acompanhamento das gestantes participantes e dos seus recém-nascidos. Maternidade Escola da Universidade Federal do Rio de Janeiro, município do Rio de Janeiro, 2021-2022 (n=844)

Considerando-se os níveis séricos de cálcio e de vitamina D nas 400 gestantes que tiveram amostras de sangue suficientes para essas análises, as seguintes médias foram observadas: para cálcio, 8,58±0,62; para vitamina D, 20,10±7,52. Desse grupo, foi constatado que 361 (90,3%) gestantes apresentaram níveis insuficientes de vitamina D, enquanto 250 (62,5%) apresentaram níveis de cálcio abaixo do normal (Tabela 1).

Embora tenha sido observado que quanto menor a circunferência craniana, menor o peso e os níveis séricos de cálcio, as correlações foram, respectivamente, fortes (r=0,805; p-valor<0,001) e fracas (r=0,129; p-valor 0,01). Para os níveis de vitamina D, não houve correlação (r=0,051; p-valor 0,310).

Foram observados valores médios menores do perímetro cefálico de recém-nascidos cujas mães consumiram álcool (p-valor 0,016) e drogas ilícitas (p-valor 0,013) durante a gravidez. Bebês do sexo feminino, prematuros, gêmeos e aqueles bebês com baixo peso ao nascer também apresentaram valores médios menores do perímetro cefálico. A Tabela 2 demonstra a diferença entre os valores médios do perímetro cefálico considerando cada variável estudada.

Quando o desfecho dicotômico investigado (perímetro cefálico<32 cm/≥32 cm) foi considerado, bebês do sexo feminino, prematuros, gêmeos e aqueles bebês com baixo peso ao nascer foram associados ao perímetro cefálico inferior a 32 cm (Tabela 3). Portanto, essas variáveis foram incluídas nos modelos finais de regressão múltipla (Tabela 4).

Observou-se que nenhum fator exposto foi associado ao perímetro cefálico inferior a 32 cm (microcefalia) em todos os modelos ajustados (Tabela 4).

**Tabela 1:** Frequência absoluta (n) e percentual (%) das características dos bebês e das gestantes do estudo. Maternidade Escola da Universidade Federal do Rio de Janeiro, município do Rio de Janeiro, 2021-2022

Variáveis	n (%)
Sexo dos bebês (n=844)	
Feminino	431 (51,1)
Masculino	411 (48,7)
Indeterminadoª	2 (0,2)
Raça/cor da pele dos bebês (n=745)	
Branca	243 (32,6)
Parda	280 (37,6)
Preta	216 (29,0)
Outras	6 (0,8)
Peso ao nascer (n=833)	
<2.500 g	107 (12,8)
≥2.500 g	726 (87,2)
Perímetro cefálico (n=821)	
<32 cm	73 (8.9)
≥32 cm	748 (91.1)
Consumo de álcool (n=809)	
Sim	332 (41,0)
Não	477 (59,0)
Uso de tabaco (n=844)	
Sim	240 (28,4)
Não	604 (71,6)
Uso de drogas (n=807)	
Sim	37 (4,6)
Não	770 (95,4)
Tipo de parto (n=843)	
Normal	359 (42,5)
Cesariana	482 (57,3)
Fórceps	2 (0,2)
Tipo de gestação (n=844)	
Único	810 (96,0)
Gêmeos	34 (4,0)
Idade gestacional (n=844)	
A termo	708 (83,9)
Prematuro	136 (16,1)
Primípara (n=809)	
Sim	293 (36,2)
Não	516 (63,8)
Níveis de cálcio (n=400)	
Abaixo	250 (62,5)
Normal	150 (37,5)
Níveis de vitamina D (n=400)	
Suficiente	39 (9,7)
Insuficiente	361 (90,3)

ªO sexo não foi identificado na sala de parto, sendo classificado como indeterminado.

**Tabela 2:** Diferença entre as médias (valores em milímetros), com respectivos desvios padrão (DP) do perímetro cefálico dos bebês, considerando as variáveis dos participantes do estudo. Maternidade Escola da Universidade Federal do Rio de Janeiro, município do Rio de Janeiro, 2021-2022

Variáveis	Perímetro cefálico (média±DP)	p-valor
Sexo do bebêª		<0,001
Feminino	33,53±2,15	
Masculino	34,22±1,82	
Raça/cor da pele do bebê		0,747
Branca	33,97±1,89	
Parda	33,84±2,16	
Preta	34,01±1,89	
Outras	34,58±1,56	
Peso ao nascer		<0,001
<2.500 g	30,47±2,40	
≥2.500 g	34,35±1,41	
Consumo de álcool		0,016
Sim	33,77±2,02	
Não	34,04±1,99	
Uso de tabaco		0,192
Sim	33,74±2,03	
Não	33,89±2,04	
Uso de drogas ilícitas		0,013
Sim	33,32±1,72	
Não	33,96±2,02	
Tipo de parto		0,196
Normal	33,71±1,80	
Cesariana	33,96±2,19	
Fórceps	33,50±0,70	
Tipo de gestação		<0,001
Única	33,95±1,98	
Gemelar	31,45±2,09	
Idade gestacional		<0,001
A termo	34,30±1,50	
Prematuro	31,39±2,68	
Primípara		0,151
Sim	33,79±1,88	
Não	34,00±2,08	

ªOs valores de perímetro cefálico dos bebês com sexo indeterminado não foram considerados nas análises estatísticas.

**Tabela 3:** Associação do perímetro cefálico (<32 cm ou ≥32 cm) dos bebês às variáveis pesquisadas, demonstradas por análise bivariada, risco relativo (RR) e intervalos de confiança de 95% (IC95%). Maternidade Escola da Universidade Federal do Rio de Janeiro, município do Rio de Janeiro, 2021-2022

Variável		Perímetro cefálico			Regressão de Poisson		
	n	<32cm	≥32cm	p-valor	Modelo bruto		
		n (%)	n (%)		RR	IC95%	p-valor
Sexo do bebê				0,006			
Masculino	406	25 (3,0)	381 (46,5)		1,056	(1,016; 1,098)	0,006
Feminino	414	48 (5,9)	366 (44,6)		1		
Raça/cor da pele do bebê				0,816			
Outra	6	-	6 (0,8)		-	-	-
Parda	275	23 (3,2)	252 (34,6)		0,980	(0,937; 1,026)	0,391
Preta	212	16 (2,2)	196 (26,9)		0,988	(0,943; 1,036)	0,630
Branca	235	15 (2,1)	220 (30,2)		1		
Peso ao nascer				<0,001			
≥2,500 g	721	12 (1,5)	709 (86,4)		1,810	(1,644; 1,993)	<0,001
<2,500 g	100	61 (7,4)	39 (4,8)		1		
Consumo de álcool				0,414			
Não	467	33 (4,2)	434 (54,9)		1,016	(0,978; 1,056)	0,422
Sim	324	28 (3,5)	296 (37,4)		1		
Uso de tabaco				0,359			
Não	589	49 (6,0)	540 (65,8)		1,020	(0,975; 1,068)	0,379
Sim	232	24 (2,9)	208 (25,3)		1		
Uso de drogas				0,189			
Não	753	56 (7,1)	697 (88,3)		1,067	(0,951; 1,196)	0,269
Sim	36	5 (0,6)	31 (3,9)		1		
Tipo de parto				1,000			
Normal	353	31(3,8)	322 (39,3)		1,003	(0,964; 1,043)	0,901
Cesariana	465	42 (5,1)	423 (51,6)		1		
Fórceps	2	-	2 (0,2)		-	-	-
Tipo de gestação				<0,001			
Gêmeos	30	14 (1,7)	16 (1,9)		0,676	(0,565; 0,808)	<0,001
Única	791	59 (7,2)	732 (89,2)		1		
Idade gestacional				<0,001			
A termo	696	21 (2,6)	675 (82,2)		1,471	(1,348; 1,605)	<0,001
Prematuro	125	52 (6,3)	73 (8,9)		1		
Primípara				0,971			
Não	504	39 (4,9)	465 (58,8)		0,999	(0,961; 1,039)	0,971
Sim	287	22 (2,8)	265 (33,5)		1		
Níveis de cálcio				0,577			
Abaixo	244	26 (6,7)	218 (55,9)		0,983	(0,925; 1,044)	0,569
Normal	146	13 (3,3)	133 (34,1)		1		
Níveis de vitamina D				0,955			
Insuficiente	351	35 (9,0)	316 (81,0)		1,003	(0,907; 1,109)	0,956
Suficiente	39	4 (1,0)	35 (9,0)		1		

**Tabela 4:** Influência dos fatores de exposição sobre os valores médios de perímetro cefálico (<32 cm/≥32 cm) dos bebês demonstrados por modelos múltiplos ajustados^a^ por: peso ao nascer (Modelo 0); sexo (Modelo 1), tipo de parto (Modelo 2) e idade gestacional (Modelo 3). Maternidade Escola da Universidade Federal do Rio de Janeiro, município do Rio de Janeiro, 2021-2022

	Modelo 0					Modelo 1				
	Total	n	RR	(IC95%)	p-valor	Total	n	RR	(IC95%)	p-valor
Vitamina D<30 ng/mL	390	351	0,981	(0,922; 1,044)	0,543	390	351	1,004	(0,910; 1,109)	0,931
Cálcio<8,8 mg/dL	390	244	0,975	(0,934; 1,019)	0,263	390	244	0,990	(0,933; 1,052)	0,753
Sem uso de álcool	791	467	1,006	(0,977; 1,035)	0,699	790	467	1,015	(0,977; 1,055)	0,444
Sem uso de tabaco	821	589	1,025	(0,990; 1,061)	0,158	820	589	1,019	(0,974; 1,066)	0,412
Sem uso de drogas	789	753	1,060	(0,969; 1,160)	0,202	788	752	1,065	(0,950; 1,194)	0,280
	Modelo 2					Modelo 3				
	Total	n	RR	(IC95%)	p-valor	Total	n	RR	(IC95%)	p-valor
Vitamina D<30 ng/mL	390	351	1,022	(0,926; 1,129)	0,662	390	351	1,007	(0,937; 1083)	0,844
Cálcio<8,8 mg/dL	390	244	0,971	(0,918; 1,027)	0,304	390	244	0,982	(0,931; 1,037)	0,523
Sem uso de álcool	791	467	1,010	(0,973; 1,049)	0,601	791	467	1,017	(0,983; 1,053)	0,351
Sem uso de tabaco	821	589	1,042	(0,997; 1,089)	0,069	821	589	1,038	(0,999; 1,079)	0,059
Sem uso de drogas	789	753	1,069	(0,954; 1,199)	0,251	789	753	1,098	(0,983; 1,226)	0,097

a Categorias de referência**:** Vitamina D≥30 ng/mL, Cálcio=8,8 a 11,0 mg/dL, uso de álcool, uso de tabaco, uso de drogas ilícitas; Modelo 0: ajustado pelo peso ao nascer, Modelo 1: ajustado pelo sexo, categoria de referência: feminino; Modelo 2: ajustado pelo tipo de parto, categoria de referência: parto de filho único; Modelo 3: ajustado pela idade gestacional, categoria de referência: a termo. Referência para o desfecho: perímetro cefálico≥32 cm. Risco relativo (RR); intervalo de confiança de 95% (IC95%).

## Discussão 

Entre os principais achados do presente estudo, observou-se que bebês nascidos de mães que consumiram álcool e drogas ilícitas, bebês do sexo feminino, bebês prematuros, bebês gêmeos e aqueles com baixo peso ao nascer apresentaram valores de perímetro cefálico mais baixos. Além disso, foi encontrada uma forte correlação positiva entre o peso do bebê e o perímetro cefálico. Estudos anteriores ^9^^,^^20^^,^^21^ também indicaram que o consumo de álcool e de drogas durante a gravidez pode afetar negativamente o desenvolvimento fetal, incluindo o perímetro cefálico, o que confirma os achados deste estudo. Ademais, a prematuridade e o baixo peso ao nascer são amplamente reconhecidos como fatores que influenciam negativamente o crescimento craniano ^21^. Porém, embora valores médios de perímetro cefálico mais baixos tenham sido observados em bebês de mães que consumiram álcool e drogas ilícitas durante a gravidez, não foi observada associação entre microcefalia (perímetro cefálico<32 cm) e tais fatores de exposição, nem com os demais fatores investigados. Como o valor médio do perímetro cefálico da amostra estudada foi acima de 32 cm e a incidência de microcefalia foi de 8,9% (73/821), sugere-se que esses resultados podem ter influenciado a falta de associação entre os fatores de exposição e o desfecho pesquisado. Ainda, a possibilidade da influência de fatores ambientais ou nutricionais não considerados na análise podem ter contribuído para a ausência de associação. 

Em relação ao uso de tabaco durante a gravidez, os resultados apresentados neste estudo indicam que não houve associação estatisticamente significativa com os valores de circunferência craniana dos recém-nascidos. No entanto, existe evidência de que o tabagismo durante a gravidez está inversamente associado ao peso, ao comprimento e à circunferência da cabeça do bebê ao nascer ^19^. Uma limitação do presente estudo é justamente o fato de não ter questionado as mães sobre a frequência e o número de cigarros consumidos, a duração da exposição ao tabaco ou o trimestre da gestação em que ocorreu o maior consumo - fatores cruciais para avaliar os efeitos do tabagismo no desenvolvimento fetal, os quais podem ter influenciado os resultados encontrados. Mesmo assim, é importante destacar que observamos uma pequena tendência (p-valor 0,059) de associação entre gestantes não fumantes e valores normais de perímetro cefálico (≥32 cm) nos casos de mães que tiveram parto prematuro (Tabela 4). Sabe-se que recém-nascidos de mulheres que fumaram após o primeiro trimestre apresentaram uma circunferência craniana menor quando comparados àqueles cujas mães pararam de fumar ^20^. Assim, acreditamos que mais estudos são necessários para esclarecer se os valores médios de perímetro cefálico, principalmente de bebês prematuros, se tornam ainda mais baixos quando as gestantes utilizam tabaco. É importante acrescentar que o uso dessas substâncias psicoativas pode comprometer a oferta de nutrientes e de oxigênio ao feto, afetando seu crescimento e desenvolvimento neurológico, por vias que envolvem alterações placentárias e sistêmicas.

Em consonância com determinado estudo ^22^, apesar de distinto quanto ao método de investigação dos micronutrientes, o presente estudo observou uma situação alarmante em relação à deficiência de vitamina D e aos baixos níveis de cálcio entre as mães participantes. Essa deficiência em grande parte da amostra destaca a magnitude do problema entre as gestantes, levantando preocupações sobre as saúdes materna e fetal. Ressalta-se que as mães foram monitoradas durante toda a gestação por meio de acompanhamento médico pré-natal. Além disso, exames laboratoriais para verificação dos níveis de vitamina D e de cálcio não são comumente solicitados para gestantes atendidas pelo Sistema Único de Saúde (SUS) no Brasil, o que levanta um alerta sobre a importância desses exames durante o pré-natal. Destacamos que a amostra pode ter apresentado viés de seleção, uma vez que participantes com acesso a serviços de saúde específicos ou com maior interesse em cuidados pré-natais podem estar desproporcionalmente representadas. Ademais, nem todas as gestantes avaliadas tiveram os níveis séricos de vitamina D e de cálcio investigados. Essa restrição pode ter introduzido um viés de seleção, considerando que as gestantes que realizaram os exames podem diferir daquelas que não os realizaram, o que influencia potencialmente os resultados. Além disso, reconhecemos que a avaliação da exposição a micronutrientes foi realizada com uma única dosagem pontual, o que pode não refletir as variações ao longo da gestação. Essas limitações podem influenciar a generalização dos resultados.

Também é preciso considerar que a ausência da quantificação do cálcio ionizado, que é a forma fisiologicamente ativa, pode ter implicações para a interpretação dos nossos resultados. Como apenas medimos o cálcio total, esse valor inclui tanto o cálcio livre quanto o ligado a proteínas, em que não se podem distinguir as variações que refletem o cálcio biodisponível. Essa limitação impacta a precisão na avaliação dos níveis de cálcio das participantes, podendo reduzir a sensibilidade em detectar associações reais entre os níveis de cálcio e os fatores estudados. Portanto, os resultados deste estudo devem ser interpretados com cautela, reconhecendo que a medição do cálcio total oferece uma visão geral, mas pode não refletir completamente o estado fisiológico do cálcio nas gestantes investigadas. Para futuras pesquisas, seria recomendável uma avaliação mais robusta do status de cálcio.

Os autores enfatizam que pessoas com pele mais escura têm proteção natural contra o sol, pois a melanina absorve a radiação UVB, e necessitam de três a cinco vezes mais exposição solar para sintetizar a mesma quantidade de vitamina D do que aquelas com pele mais clara ^23^. Observa-se que, no presente estudo, a maioria dos bebês era de raça/cor da pele parda ou negra, com suas mães apresentando as mesmas características. Essa poderia ser uma razão para os resultados encontrados, uma vez que a síntese de vitamina D depende da exposição solar. Outra razão poderia ser a inadequação alimentar, conforme sugerido em estudo ^22^ que observou baixo consumo de nutrientes importantes como o cálcio e a vitamina D por gestantes de duas coortes, independente do trimestre da gestação. Além disso, tal investigação enfatizou que, independentemente da raça/cor da pele e do uso de suplementação vitamínica, promover uma alimentação equilibrada deve ser uma prioridade desde o início do pré-natal, orientação que deve ser preservada ao longo de toda a gestação. Nesse sentido, são necessários novos estudos envolvendo a avaliação conjunta da alimentação, da exposição solar e de níveis séricos de vitamina D em gestantes.

A correlação positiva observada neste estudo entre baixos níveis séricos de cálcio materno e menores medições de circunferência craniana, embora fraca, sugere que o metabolismo mineral materno pode afetar o desenvolvimento ósseo do bebê, corroborando estudo prévio ^8^. No entanto, em relação aos níveis séricos de vitamina D, nenhuma correlação foi encontrada com as médias de circunferência craniana dos recém-nascidos, ao contrário de alguns estudos que indicam que a insuficiência de vitamina D é um fator crítico para o desenvolvimento ósseo fetal ^24^^-^^26^. A insuficiência de nutrientes essenciais, como cálcio e fósforo, somados a fatores demográficos (idade, sexo, raça/cor da pele) e ambientais (exposição ao sol), pode modular a deficiência de vitamina D e a sua ação biológica ^27^. Tais interações complexas podem influenciar a mineralização óssea e, consequentemente, mascarar os efeitos da vitamina D no crescimento craniano. A ausência de correlação encontrada neste estudo não invalida o papel da vitamina D no desenvolvimento fetal, mas enfatiza a necessidade de investigações mais aprofundadas para elucidar a complexidade dessas relações. 

Os resultados do presente estudo são relevantes e apontam para uma lacuna importante no cuidado pré-natal, pois identificar fatores de risco é crucial para a formulação de estratégias preventivas e de apoio durante a gravidez. Portanto, embora os valores de perímetro cefálico encontrados não tenham sido caracterizados como de bebês com microcefalia, este estudo destaca que tais medidas foram mais baixas nos recém-nascidos cujas mães consumiram álcool e drogas ilícitas durante a gestação. Estudos prévios ^28^^,^^29^ sugerem que um perímetro cefálico menor ao nascimento pode estar associado a um risco aumentado de assimetrias cranianas posteriores, destacando a relevância dessa medida como marcador precoce do crescimento e da conformação craniana. Além disso, a correlação encontrada entre os níveis de cálcio materno e a menor circunferência craniana dos bebês, embora fraca, enfatiza a importância de um cuidado pré-natal adequado e de medidas preventivas durante a gravidez.

## Data Availability

O banco de dados e os códigos de análise utilizados na pesquisa estão disponíveis por meio do acesso ao link: https://data.scielo.org/dataverse/scielodata?q=&fq1=publicationStatus%3A%22Draft%22&fq0=dvObjectType%3A%28dataverses+OR+datasets%29&types=dataverses%3Adatasets&sort=dateSort&order=.
